# Supplementary material for: Dendritic Cells in Anticancer Vaccination: Rationale for Ex Vivo Loading or In Vivo Targeting
Source: Cancers (Basel). 2020 Mar 5;12(3):590. doi: 10.3390/cancers12030590 (PMC7139354; doi:10.3390/cancers12030590)
Supplement: Supplementary file 1 [file cancers-12-00590-s001.pdf]

Table S1. Clinical trials with published information about the method of DC-based vaccine generation and immune and/or clinical response.

IN VIVO TARGETING									
**Type of vaccine**		**Combinatorial treatment**	**Condition**	**Trial phase**	**Detected immune response**	**Median OS, months**	**Median PFS, months**	**Clinical trial ID**	**Refs**
**Target DC**	**Targeting method**								
GM-CSF recruited DCs	Administration of irradiated GM.CD40L bystander cells, irradiated CCL21-expressing NCI-H1944 cells, irradiated NCI-H2122 cells	GM-CSF	Lung adenocarcinoma	I/II	N/S	9.5	3.4	NCT01433172	[1]
GM-CSF recruited DCs	Administration of irradiated GM.CD40L bystander cells, irradiated NCI-H1944 and NCI-H2122 cells	CYP	Refractory lung adenocarcinoma	II	N/A	23 (7.1)	N/S	NCT00601796	[2]
	Administration of synthetic HLA-restricted peptides, corresponding to personal neoantigens	TLR3, Poly(IC:LC)	Melanoma	I	$CD4^+$ T cells; $CD8^+$ T cells	N/A	N/A	NCT01970358	[3]
GM-CSF recruited DCs	Administration of multiepitope vaccine, composed of tyrosinase, gp100, MART-1 peptides	GM-CSF	Melanoma	III	$CD8^+$ T cells	68.6 (63.3) N/S	11.5 (9.8) N/S	NCT01989572	[4,5]
Secondary lymphoid organ-resident DCs targeting	Administration of autologous OKT3, IL-2 PBMC-derived lymphocytes, transduced with retroviral LM3TN vector, encoding MAGE-A3 and HSV-TKneo	N/U	Melanoma	II	T-cell response; DTH-positive test	N/R (12)	N/A	Not available	[6]
	Administration of MM200 cell line derived liposomes, comprised of: MM200 derived 3NTA-DTDA/ POPC modified plasma membrane vesicles containing gp100, tyrosinase and MART-1; IFNγ; DC-SIGN-specific domain Ab DMS5000	N/U	Melanoma	I	N/S	N/A	N/A	Not available	[7]
GM-CSF recruited DCs	Administration of irradiated GM.CD40L bystander cells and irradiated autologous tumor cells	GM-CSF	Different resectable tumors	I	$CD4^+$ T cells; $CD8^+$ T cells	N/A	N/A	Not available	[8]
	Administration of MVA-brachyury-TRICOM vaccine (MVA strain of vaccinia encoding expression of brachyury, B7.1, LFA-3, ICAM-1	N/U	Different advanced cancers	I	$CD4^+$ T cells; $CD8^+$ T cells	N/A	N/A	NCT02179515	[9]
GM-CSF recruited DCs	Administration of CDX-1307 fusion protein, comprised of MR-specific IgG_1 and hCG-β	GM-CSF, Poly(IC:LC), resiquimod	Different advanced cancers	I	T-cell response; humoral	N/A	N/A	NCT00709462; NCT00648102	[10]
	Administration of CDX-1401 fusion protein, comprised of anti-DEC-205 monoclonal antibody fused to the full-length NY-ESO-1	Poly(IC:LC), resiquimod	Different advanced cancers	I	$CD4^+$ T cells; $CD8^+$ T cells; IFN-γ-producing cells; humoral	N/A	N/A	NCT00948961	[11]
	Administration of autologous tumor-derived HSP96-peptide complexes	CYP, TMZ	Glioblastoma	I	IFN-γ-producing cells	31.4	11	NCT02122822; ChiCTR-ONC-13003309	[12]
	Administration of autologous tumor-derived HSP96-peptide complexes	Radiotherapy, TMZ	Glioblastoma	II	N/A	23.8	18	NCT00905060	[13]
	Administration of autologous tumor-derived HSP96-peptide complexes	N/U	Glioblastoma	II	N/A	42.6	19.1	NCT00293423	[14]

	Administration of HspE7, which is *M. bovis* HSP65 fused to HPV 16 E7 protein	N/U	Cervical intraepithelial neoplasia III	II	humoral	N/A	N/A	NCT00075569	[15,16]
	Administration of autologous tumor-derived HSP96-peptide complexes	GM-CSF, IFN-α	Melanoma	II	IFN-γ-producing cells	N/A	N/A	Not available	[17]
	Administration of autologous tumor-derived HSP96-peptide complexes	N/U	Pancreatic adenocarcinoma	I	DTH-positive test; IFN-γ-producing cells; CD8$^+$ T cells;	2.2	0.9	Not available	[18]
	Administration of LV305, which is NY-ESO-1-encoding lentiviral vector, functionalized to target DC-SIGN	N/U	Different NY-ESO-1-expressing tumors	I	CD4$^+$ T cells; CD8$^+$ T cells; humoral	31.1	4.6	NCT02122861	[19]
**EX VIVO LOADING**									
**Type of vaccine**		**Combinatorial treatment**	**Condition**	**Trial phase**	**Detected immune response**	**Median OS, months**	**Median PFS, months**	**Clinical trial ID**	**Refs**
**DCs source**	**Loading method**								
GM-CSF, IL-4 autologous moDCs	Loading with MART-1 peptide	Tremelimumab	Melanoma	I	CD4$^+$ T cells; CD8$^+$ T cells	N/A	N/A	NCT00090896	[20]
GM-CSF, IL-4 autologous moDCs	Loading with HLA-restricted melanoma-specific peptides or with autologous tumor lysate + KLH	N/U	Melanoma		DTH-positive test	N/A	N/A	Not available	[21]
GM-CSF, IL-4 autologous moDCs	PEG-mediated fusion with autologous irradiated tumor cells	IL-2	Melanoma	I/IIa	N/A	16.1 (8.5)	N/A	Not available	[22]
GM-CSF, IL-13 autologous moDCs	Loading with allogeneic M17 cell line lysate	N/U	Melanoma	I/II	CD8$^+$ T cells	N/A	N/A	Not available	[23]
GM-CSF, IFN-α2b autologous moDCs	N/U	Dacarbazine	Melanoma	I	CD8$^+$ T cells	N/A	N/A	Not available	[24]
GM-CSF, IL-4 autologous moDCs; matured with TNF-α	Loading with Mage-3A1 peptide	N/U	Melanoma		CD8$^+$ T cells	N/A	N/A	Not available	[25]
GM-CSF, IL-4 autologous moDCs; matured with TNF-α	Loading with autologous tumor lysate	CYP, ATTL	Melanoma	I	T-cell response	20 (10)	N/A	Not available	[26]
GM-CSF, IL-4 autologous moDCs; matured with IL-1β, IL-6, TNF-α, PGE2	Loading with HLA-restricted melanoma-specific peptides	N/U	Melanoma	I	CD4$^+$ T cells; DTH-positive test	N/A	N/A	Not available	[27]
GM-CSF, IL-4 autologous moDCs; matured with PGE2, OK432	Loading with synthetic HLA-restricted WT, gp100, tyrosinase, MAGE-A2 peptides	Carboplatin, paclitaxel	Melanoma	I/II	CD8$^+$ T cells; IFN-γ-producing cells	12	2.3	UMIN000006629	[28]
GM-CSF, IL-4 autologous moDCs; matured with TNF-α, PGE2	Loading with gp100, tyrosinase peptides + KLH	IL-2	Melanoma	I/II	CD8$^+$ T cells; B-cells; humoral; DTH-positive test	N/A	N/A	NCT00243594	[29,30]
GM-CSF, IL-4 autologous moDCs; matured with IL-1β, IL-6, TNF-α, PGE2, α-GalCer	Loading with two NY-ESO-1 peptides, peptides from influenza virus proteins (PB-1, M1, NP)	N/U	Melanoma	I	CD4$^+$ T cells; CD8$^+$ T cells; NK cells	N/A	N/A	Not available	[31]
GM-CSF, IL-4 autologous moDCs; matured with TNF-α, PGE2	Loading with gp-100 and tyrosinase peptides, or electroporation with full length-encoding gp-100 and tyrosinase mRNA	IL-2	Melanoma	III	T-cell response	63.6 (31)	41.9 (24.3)	Not available	[32]
GM-CSF, IL-4 autologous moDCs; matured with TNF and CD40L	Loading with allogeneic apoptotic and necrotic Colo829 cells	N/U	Melanoma	I	CD8$^+$ T cells	22.5	N/A	Not available	[33]

GM-CSF, IL-4 autologous moDCs; matured by electroporation with poly I:C12U and CD40L mRNA, or with CD40L, CD70 and caTLR4 mRNA	Electroporation with 1 out of listed antigen-coding mRNA: MAGE-A1, MAGEA3, MAGE-C2, MelanA/MART-1, tyrosinase, or gp100	IFN-α-2b	Melanoma	I/II	$CD8^+$ T cells; DTH-positive test	N/R	22	NCT00074230; NCT01066390	[34-36]
Electroporation of autologous $CD14^+$ cells with siRNAs targeting 3 inducible immunoproteasome subunits; GM-CSF, IL-4 autologous moDCs; matured with IL-1β, IL-6, TNF-α, PGE2	Electroporation with mRNAa encoding MART-1, MAGE-3,TYR, gp100	N/U	Melanoma	I	$CD4^+$ T cells; $CD8^+$ T cells	N/A	N/A	NCT00672542	[37]
IL-3, IL-6, SCF, IL-4, GM-SCF autologous $CD34^+$ precursors; matured with TNF-α	Loading with HLA-restricted melanoma-specific peptides	N/U	Melanoma	I	$CD4^+$ T cells; $CD8^+$ T cells; DTH-positive test	N/A	N/A	Not available	[38]
GM-CSF, FLT3-L, TNF autologous $CD34^+$ precursors	Loading with HLA-restricted melanoma-specific peptides + KLH	N/U	Melanoma		$CD8^+$ T cells; DTH-positive test	N/A	N/A	Not available	[39]
Autologous peripheral blood $CD1c^+$ isolated myeloid DCs; cultured with GM-SCF, KLH	Loading with HLA-restricted melanoma-specific peptides	N/U	Melanoma	I	$CD8^+$ T cells	29 (10.9)	17.6 (2.3)	NCT01690377	[40]
GM-CSF, IL-4 autologous moDCs; matured with TNF-α, PGE2	Coculture with apoptotic LNCaP	N/U	Prostate cancer	I/II	$CD4^+$ T cells; $CD8^+$ T cells	N/A	N/A	NCT00289341	[41,42]
GM-CSF, IL-4 autologous moDCs; matured with TNF-α, PGE2	Coculture with apoptotic PC3	N/U	Prostate cancer	I/II	$CD4^+$ T cells; $CD8^+$ T cells	N/A	N/A	NCT00345293	[41,42]
GM-CSF, IL-4 autologous moDCs; matured with Poly I:C	Loading with irradiated LNCaP cell line lysate	docetaxel, prednisone, CYP, imiquimod	Prostate cancer	I/II	$CD3^+$/HLA-DR^+; $CD8^+$ T cells; humoral	19 (11.8/13)	N/A	EudraCT 2009-017295-24	[43]
GM-CSF, IL-4 autologous moDCs; matured with Poly I:C	Loading with irradiated LNCaP cell line lysate	CYP, imiquimod	Prostate cancer	I/II	$CD8^+$ T cells; humoral	N/A	N/A	EudraCT 2009-017259-91	[44]
GM-CSF, IL-4 autologous moDCs; matured with IL-1β, IL-6, TNF-α, PGE2	Transfection with PAP and PSA coding mRNA	Docetaxel	Prostate cancer	II	IFN-γ-producing cells; DTH-positive test	N/A	5.7 (5.5)	NCT01446731	[45]
GM-CSF, IL-4 autologous moDCs; matured with LPS, rimiducid	Transduction with Ad5f35 adenovirus vector encoding ihCD40	Rimiducid	Prostate cancer	I	$CD8^+$ T cells	N/A	N/A	Not available	[46]
Autologous Peripheral blood APCs fraction	Loading with PAP fused with GM-CSF	N/U	Prostate cancer	III	T-cell response	25.9 (21.4)	11.7 (10)	NCT00005947	[47]
Autologous peripheral blood APCs fraction	Loading with PAP fused with GM-CSF	N/U	Prostate cancer	III	T-cell response; humoral	25.8 (21.7)	14.4 (14.6)	NCT00065442	[48]
Autologous peripheral blood $CD1c^+$ isolated myeloid DCs;	Loading with synthetic HLA-restricted PSA, PSMA, PAP, FMP + KLH	N/U	Prostate cancer	I	DTH-positive test	18	N/A	Not available	[49]
GM-CSF, IL-4 autologous moDCs; matured with TNF-α, PGE2	Coculture with autologous apoptotic brain tumor cells + KLH	N/U	Primary brain cancer	I	$CD4^+$ T cells; $CD8^+$ T cells	N/A	N/A	NCT00893945	[41]
GM-CSF, IL-4 autologous moDCs	Coculture with autologous irradiated tumor cells	TMZ	Glioblastoma	I	N/S	23	N/A	ACTRN 12611000029998	[50]
GM-CSF, IL-4 autologous moDCs	Loading with autologous irradiated glioma stem cell lysate	Radiotherapy or chemotherapy	Glioblastoma	II	$CD4^+$ T cells; $CD8^+$ T cells	13.7 (10.7)	7.7 (6.9)	Not available	[51]
GM-CSF, IL-4 autologous moDCs; matured with TNF-α	Loading with synthetic HLA-restricted tumor-specific peptides	N/U	Glioblastoma	I	$CD8^+$ T cells	38.4	16.9	Not available	[52]
GM-CSF, IL-4 autologous moDCs; mature with TNF-α , IL-1β, IL-6	Electroporation with CMV pp65-mRNA	Adoptive T-cell transfer	Glioblastoma	I	$CD8^+$ T cells	N/A	N/A	NCT00693095	[53]

GM-CSF, IL-4 autologous moDCs; matured with TNF-α, IL-1β, PGE2	Loading with autologous tumor lysate derived proteins	TMZ, radiotherapy	Glioblastoma	II	$CD4^+$ T cells; $CD8^+$ T cells; NK cells	19	N/A	EudraCT 2009-018228-14	[54]
GM-CSF, IL-4 autologous moDCs; matured with TNF-α, IL-1β, PGE2	Loading with autologous tumor lysate	TMZ, radiotherapy	Glioblastoma	I/II	N/S	19.4	11	EudraCT 2006-002881-20	[55]
autologous moDCs; matured with TNF-α, PGE2	Loading with autologous tumor lysate	TMZ	Glioblastoma	II	$CD4^+$ T cells	28	9.5	NCT00323115	[56]
GM-CSF, IL-4 autologous moDCs; matured with TNF-α, IFN-α, poly I:C	Loading with autologous tumor lysate	Surgery, radiotherapy, TMZ	Glioblastoma	II	$CD4^+$ T cells; IFN-γ-producing cells	23.4	12.7	NCT01006044; EudraCT 2009-009879-35	[57]
GM-CSF, TNF autologous $CD34^+$ precursors	Transfection with pp65-LAMP/A64 mRNA	TMZ, tetanus/diphtheria toxoid	Glioblastoma	I	N/A	N/R (18.5)	N/R (10.8)	NCT00639639	[58]
GM-CSF, TNF autologous $CD34^+$ precursors	Transfection with pp65-encoding mRNA	TMZ, GM-CSF	Glioblastoma	I	$CD8^+$ T cells	41.1 (8)	25.3 (19.2)	NCT00639639	[59]
GM-CSF, IL-4 autologous moDCs	Coculture with apoptosed-irradiated 1650 cell line	N/U	Non-small-cell lung carcinoma	II	IFN-γ-producing cells	N/A	N/A	NCT00103116	[60,61]
GM-CSF, IL-4 autologous moDCs	Transduction with adenovirus vector encoding CCL21	N/U	Non-small-cell lung carcinoma	I	$CD8^+$ T cells; humoral	3.9	N/A	NCT01574222	[62]
GM-CSF, IL-4 autologous moDCs; matured with TNF-α	Loading with MUC-1 peptide	CIK, radiotherapy	Non-small-cell lung carcinoma		N/S	13.3 (15.3)	11 (7.7)	ChiCTR-TRC-12002369	[63]
GM-CSF, IL-4 autologous moDCs; matured with TNF-α	Loading with allogeneic A549 or SK-MES- cell lines lysate	CIK	Non-small-cell lung carcinoma		$CD8^+$ T cells	N/A	N/A	Not available	[64]
GM-CSF, IL-4 autologous moDCs; matured with iAPA, flagellin	Loading with surviving and MUC1 peptides, and SOCS1-specific siRNA	N/U	Non-small-cell lung carcinoma	I	Increase in cytokine levels	N/A	N/A	Not available	[65]
Isolation of DCs and ATK from tumor draining lymph nodes	N/U	Platinum-based chemotherapy, adoptive transfer of DCs/ATK mixture	Non-small-cell lung carcinoma	III	N/A	N/R (47.5)	N/R (16.56)	Not available	[66]
GM-CSF, IL-4 autologous moDCs	Transduction with adenovirus vector encoding p53	All-trans-retinoic acid	Small cell lung cancer	II	IFN-γ-producing cells	N/S	N/S	Not available	[67]
GM-CSF, IL-4 autologous moDCs	Loading with autologous tumor stem cell lysate	N/U	Hepatocellular carcinoma	I	N/A	N/A	N/A	Not available	[68]
GM-CSF, IL-4 autologous moDCs; matured with TNF-α	Electroporation with HSP70 mRNA	N/U	Hepatocellular carcinoma	I	$CD8^+$ T cells; NK cells	N/A	N/A	Not available	[69]
GM-CSF, IL-4 autologous moDCs; matured with IL-1β, IL-6, TNF-α, PGE2, IFN-γ, OK432, poly I:C	Loading with recombinant AFP, MAGE-1, and GPC-3 antigens	Imiquimod	Hepatocellular carcinoma	I/IIa	T-cell response	N/A	38.4 (10.8)	ICTRP KCT0000427	[70]
GM-CSF, IL-4 autologous moDCs	Loading with one of HLA-restricted synthetic peptides: Cap1-6D, hTERT, or surviving peptide	Poly(IC:LC)	Pancreatic cancer	I	T-cell response	7.7	3	NCT01410968	[71]
GM-CSF, IL-4 autologous moDCs; matured with PGE2, OK432	Loading with HLA-restricted WT1 peptides	Gemcitabine	Pancreatic cancer	I	DTH-positive test	N/A	N/A	Not available	[72]
GM-CSF, IL-4 autologous moDCs; matured with PGE2, OK432	Loading with synthetic HLA-restricted WT1 peptide	Gemcitabine	Pancreatic cancer	I	$CD8^+$ T cells; DTH-positive test	8.1	N/A	UMIN000004855	[73]
IL-4, GM-SCF autologous $CD34^+$ precursors; matured with TNF-α	N/U	CIK, S-1	Pancreatic cancer	I/II	CD4+ T cells	7 (4.7)	4.5 (3)	NCT01781520	[74]
GM-CSF, IL-4 allogeneic moDCs; matured with TNF-α	N/U	Allogeneic lymphocyte infusion	Hematologic cancers	I	T-cell stimulation in MLR	N/A	N/A	NCT00476177	[75]

Autologous moDCs	N/U	Rituximab, radiotherapy	Follicular lymphoma		$CD4^+$ T cells; $CD8^+$ T cells	N/A	N/A	Not available	[76]
GM-CSF, IL-4 autologous moDCs; matured with TNF-α	Loading with α-Gal–conjugated autologous lymphoma cell membranes	mDC coculture and administration with CIK	B-cell lymphoma	I	$CD4^+$ T cells; $CD8^+$ T cells; NK cells	N/A	N/A	ANZCTR 12611000988954	[77]
GM-CSF, IL-4 autologous moDCs; matured with TNF-α, PGE2	Electroporation with WT1-encoding mRNA, or WT1-DC-LAMP-encoding mRNA	N/U	Acute myeloid leukemia	II	$CD8^+$ T cells	N/R (21.1)	59.6 (8.7)	NCT00965224	[78]
GM-CSF, IL-4 autologous moDCs; matured with IL-1β, IL-6, TNF-α, PGE2	Electroporation with mRNA encoding hTERT and LAMP-1	N/U	Acute myeloid leukemia	II	IFN-γ-producing T cells; DTH-positive test	N/A	N/A	NCT00510133	[79]
GM-CSF, TNF-α, IL-4 DCOne cell line; matured with IL-1β, TNF-α, PGE2; irradiated	N/U	N/U	Acute myeloid leukemia	I	IFN-γ-producing T cells; $CD4^+$ T cells; $CD8^+$ T cells; DTH-positive test	36	N/A	Not available	[80]
GM-CSF, IL-4 autologous moDCs; matured with IL-1β, IL-6, TNF-α, PGE2	Loading with mHag peptide, or KLH	Donor lymphocyte infusion	Multiple myeloma	I/II	$CD8^+$ T cells	N/A	3.5	EudraCT 2012-002435-28	[81,82]
GM-CSF, IL-4 autologous moDCs	Loading with fusion protein, consists from oxidized mannan, MUC1 fragment, and glutathione S-transferase	N/U	Epithelial ovarian cancer	II	$CD4^+$ T cells; $CD8^+$ T cells	N/R (26)	>12.91 (4.94)	NCT01068509	[83]
GM-CSF, IL-4 autologous moDCs; matured with TNF-α	Loading with autologous tumor lysate + KLH	IL-2	Ovarian cancer	I/II	IFN-γ-producing T cells; NK cells	N/A	N/A	ICTRP KCT0000831	[84]
GM-CSF, IL-4 autologous moDCs; matured with IL-1β, IL-6, TNF-α, PGE2	Loading with recombinant HPV 16 E7 or HPV 18 E7 oncoprotein	N/U	Cervical cancer		T-cell response; humoral	N/A	N/A	Not available	[85]
GM-CSF, IL-4 autologous moDCs; matured with IFN-γ, LPS, IL-1β, IL-6, TNF-α, PGE2	HER2/neu peptides loading	N/U	Breast cancer	I	$CD4^+$ T cells; $CD8^+$ T cells	N/A	N/A	NCT02063724	[86]
GM-CSF, IL-4 autologous moDCs; matured with IFN-γ, LPS	Loading with HER2 HLA-restricted peptides	N/U	Ductal carcinoma	I/II	$CD4^+$ T cells; $CD8^+$ T cells	N/A	N/A	NCT02061332	[87]
GM-CSF, IL-4 autologous moDCs; matured with TNF-α	Loading with autologous tumor lysate	IL-2, mDC coculture and administration with CIK	Renal cell carcinoma		$CD4^+$ T cells; $CD8^+$ T cells	28 (11)	N/A	Not available	[88]
GM-CSF, IL-4 allogeneic moDCs; matured with IFN-γ, poly I:C, R848	N/U	Surgery	Renal cell carcinoma	I/II	$CD4^+$ T cells; $CD8^+$ T cells	N/R	N/A	NCT01525017	[89]
GM-CSF, IL-4 autologous moDCs; matured with IL-1β, IL-6, TNF-α, PGE2	Loading with MAGE-A1, MAGE-A3, NY-ESO-1 peptides	Imiquimod	Neuroblastoma, sarcoma	I	$CD4^+$ T cells; $CD8^+$ T cells	N/A	N/A	NCT01241162	[90]
GM-CSF, IL-4 autologous moDCs; matured TNF-α, OK432	Loading with autologous tumor lysate	N/U	Bone and soft tissue sarcoma	I/II	IFN-γ-producing cells	N/A	N/A	Not available	[91]
GM-CSF, IL-4 autologous moDCs; matured with TNF-α, KLH, OK432	Loading with synthetic HLA-restricted RNF43 peptides	DC-activated autologous killer lymphocytes, IL-2, CYP	Different advanced solid tumors	I	$CD8^+$ T cells	N/A	N/A	UMIN000003945	[92]

Abbreviations used in table: Ab - antibody; ATK – activated T killers; ATTL – adoptive transfer of tumor-infiltrated lymphocytes; CIK – cytokine-induced killer cells; CYP – cyclophosphamide; DTH - delayed-type hypersensitivity reaction; DTIC – dacarbazine; MLR – mixed leukocyte reaction; moDCs - monocyte-derived dendritic cells; N/A – not assessed; N/R – not reached due to only few deaths of patients; N/S – not significant; N/U – was not used; Poly I:C, poly I:C_{12}U, Poly(IC:LC) - polyinosinic-polycytidylic acid and its different derivatives; S-1 – combination drug

comprised of fluoropyrimidine tegafur, DPD-inhibitor gimeracil, and oteracil potassium; TMZ – temozolomide; α-Gal - α-1,3-galactosyltransferase. Control, non-responders, or other comparison group numbers are indicated in brackets if available.

1. Gray, J.E.; Chiappori, A.; Williams, C.C.; Tanvetyanon, T.; Haura, E.B.; Creelan, B.C.; Kim, J.; Boyle, T.A.; Pinder-Schenck, M.; Khalil, F., et al. A phase I/randomized phase II study of GM.CD40L vaccine in combination with CCL21 in patients with advanced lung adenocarcinoma. *Cancer immunology, immunotherapy : CII* **2018**, *67*, 1853-1862, doi:10.1007/s00262-018-2236-7.
2. Creelan, B.C.; Antonia, S.; Noyes, D.; Hunter, T.B.; Simon, G.R.; Bepler, G.; Williams, C.C.; Tanvetyanon, T.; Haura, E.B.; Schell, M.J., et al. Phase II trial of a GM-CSF-producing and CD40L-expressing bystander cell line combined with an allogeneic tumor cell-based vaccine for refractory lung adenocarcinoma. *J Immunother* **2013**, *36*, 442-450, doi:10.1097/CJI.0b013e3182a80237.
3. Ott, P.A.; Hu, Z.; Keskin, D.B.; Shukla, S.A.; Sun, J.; Bozym, D.J.; Zhang, W.; Luoma, A.; Giobbie-Hurder, A.; Peter, L., et al. An immunogenic personal neoantigen vaccine for patients with melanoma. *Nature* **2017**, *547*, 217-221, doi:10.1038/nature22991.
4. Lawson, D.H.; Lee, S.; Zhao, F.; Tarhini, A.A.; Margolin, K.A.; Ernstoff, M.S.; Atkins, M.B.; Cohen, G.I.; Whiteside, T.L.; Butterfield, L.H., et al. Randomized, Placebo-Controlled, Phase III Trial of Yeast-Derived Granulocyte-Macrophage Colony-Stimulating Factor (GM-CSF) Versus Peptide Vaccination Versus GM-CSF Plus Peptide Vaccination Versus Placebo in Patients With No Evidence of Disease After Complete Surgical Resection of Locally Advanced and/or Stage IV Melanoma: A Trial of the Eastern Cooperative Oncology Group-American College of Radiology Imaging Network Cancer Research Group (E4697). *Journal of clinical oncology : official journal of the American Society of Clinical Oncology* **2015**, *33*, 4066-4076, doi:10.1200/JCO.2015.62.0500.
5. Butterfield, L.H.; Zhao, F.; Lee, S.; Tarhini, A.A.; Margolin, K.A.; White, R.L.; Atkins, M.B.; Cohen, G.I.; Whiteside, T.L.; Kirkwood, J.M., et al. Immune Correlates of GM-CSF and Melanoma Peptide Vaccination in a Randomized Trial for the Adjuvant Therapy of Resected High-Risk Melanoma (E4697). *Clinical cancer research : an official journal of the American Association for Cancer Research* **2017**, *23*, 5034-5043, doi:10.1158/1078-0432.CCR-16-3016.
6. Russo, V.; Pilla, L.; Lunghi, F.; Crocchiolo, R.; Greco, R.; Ciceri, F.; Maggioni, D.; Fontana, R.; Mukenge, S.; Rivoltini, L., et al. Clinical and immunologic responses in melanoma patients vaccinated with MAGE-A3-genetically modified lymphocytes. *International journal of cancer* **2013**, *132*, 2557-2566, doi:10.1002/ijc.27939.
7. Gargett, T.; Abbas, M.N.; Rolan, P.; Price, J.D.; Gosling, K.M.; Ferrante, A.; Ruszkiewicz, A.; Atmosukarto, I.I.C.; Altin, J.; Parish, C.R., et al. Phase I trial of Lipovaxin-MM, a novel dendritic cell-targeted liposomal vaccine for malignant melanoma. *Cancer immunology, immunotherapy : CII* **2018**, *67*, 1461-1472, doi:10.1007/s00262-018-2207-z.
8. Dessureault, S.; Noyes, D.; Lee, D.; Dunn, M.; Janssen, W.; Cantor, A.; Sotomayor, E.; Messina, J.; Antonia, S.J. A phase-I trial using a universal GM-CSF-producing and CD40L-expressing bystander cell line (GM.CD40L) in the formulation of autologous tumor cell-based vaccines for cancer patients with stage IV disease. *Annals of surgical oncology* **2007**, *14*, 869-884, doi:10.1245/s10434-006-9196-4.
9. Heery, C.R.; Palena, C.; McMahon, S.; Donahue, R.N.; Lepone, L.M.; Grenga, I.; Dirmeier, U.; Cordes, L.; Marte, J.; Dahut, W., et al. Phase I Study of a Poxviral TRICOM-Based Vaccine Directed Against the Transcription Factor Brachyury. *Clinical cancer research : an official journal of the American Association for Cancer Research* **2017**, *23*, 6833-6845, doi:10.1158/1078-0432.CCR-17-1087.
10. Morse, M.A.; Chapman, R.; Powderly, J.; Blackwell, K.; Keler, T.; Green, J.; Riggs, R.; He, L.Z.; Ramakrishna, V.; Vitale, L., et al. Phase I study utilizing a novel antigen-presenting cell-targeted vaccine with Toll-like receptor stimulation to induce immunity to self-antigens in cancer patients. *Clinical cancer research : an official journal of the American Association for Cancer Research* **2011**, *17*, 4844-4853, doi:10.1158/1078-0432.CCR-11-0891.
11. Dhodapkar, M.V.; Sznol, M.; Zhao, B.; Wang, D.; Carvajal, R.D.; Keohan, M.L.; Chuang, E.; Sanborn, R.E.; Lutzky, J.; Powderly, J., et al. Induction of antigen-specific immunity with a vaccine targeting NY-ESO-1 to the dendritic cell receptor DEC-205. *Sci Transl Med* **2014**, *6*, 232ra251, doi:10.1126/scitranslmed.3008068.
12. Ji, N.; Zhang, Y.; Liu, Y.; Xie, J.; Wang, Y.; Hao, S.; Gao, Z. Heat shock protein peptide complex-96 vaccination for newly diagnosed glioblastoma: a phase I, single-arm trial. *JCI Insight* **2018**, *3*, doi:10.1172/jci.insight.99145.
13. Bloch, O.; Lim, M.; Sughrue, M.E.; Komotar, R.J.; Abrahams, J.M.; O'Rourke, D.M.; D'Ambrosio, A.; Bruce, J.N.; Parsa, A.T. Autologous Heat Shock Protein Peptide Vaccination for Newly Diagnosed Glioblastoma: Impact of Peripheral PD-L1 Expression on Response to Therapy.

Clinical cancer research : an official journal of the American Association for Cancer Research **2017**, *23*, 3575-3584, doi:10.1158/1078-0432.CCR-16-1369.
14. Bloch, O.; Crane, C.A.; Fuks, Y.; Kaur, R.; Aghi, M.K.; Berger, M.S.; Butowski, N.A.; Chang, S.M.; Clarke, J.L.; McDermott, M.W., et al. Heat-shock protein peptide complex-96 vaccination for recurrent glioblastoma: a phase II, single-arm trial. *Neuro Oncol* **2014**, *16*, 274-279, doi:10.1093/neuonc/not203.
15. Einstein, M.H.; Kadish, A.S.; Burk, R.D.; Kim, M.Y.; Wadler, S.; Streicher, H.; Goldberg, G.L.; Runowicz, C.D. Heat shock fusion protein-based immunotherapy for treatment of cervical intraepithelial neoplasia III. *Gynecol Oncol* **2007**, *106*, 453-460, doi:10.1016/j.ygyno.2007.04.038.
16. Van Doorslaer, K.; Reimers, L.L.; Studentsov, Y.Y.; Einstein, M.H.; Burk, R.D. Serological response to an HPV16 E7 based therapeutic vaccine in women with high-grade cervical dysplasia. *Gynecol Oncol* **2010**, *116*, 208-212, doi:10.1016/j.ygyno.2009.05.044.
17. Pilla, L.; Patuzzo, R.; Rivoltini, L.; Maio, M.; Pennacchioli, E.; Lamaj, E.; Maurichi, A.; Massarut, S.; Marchiano, A.; Santantonio, C., et al. A phase II trial of vaccination with autologous, tumor-derived heat-shock protein peptide complexes Gp96, in combination with GM-CSF and interferon-alpha in metastatic melanoma patients. *Cancer immunology, immunotherapy : CII* **2006**, *55*, 958-968, doi:10.1007/s00262-005-0084-8.
18. Maki, R.G.; Livingston, P.O.; Lewis, J.J.; Janetzki, S.; Klimstra, D.; Desantis, D.; Srivastava, P.K.; Brennan, M.F. A phase I pilot study of autologous heat shock protein vaccine HSPPC-96 in patients with resected pancreatic adenocarcinoma. *Dig Dis Sci* **2007**, *52*, 1964-1972, doi:10.1007/s10620-006-9205-2.
19. Somaiah, N.; Block, M.S.; Kim, J.W.; Shapiro, G.I.; Do, K.T.; Hwu, P.; Eder, J.P.; Jones, R.L.; Lu, H.; Ter Meulen, J.H., et al. First-in-Class, First-in-Human Study Evaluating LV305, a Dendritic-Cell Tropic Lentiviral Vector, in Sarcoma and Other Solid Tumors Expressing NY-ESO-1. *Clinical cancer research : an official journal of the American Association for Cancer Research* **2019**, *25*, 5808-5817, doi:10.1158/1078-0432.CCR-19-1025.
20. Ribas, A.; Comin-Anduix, B.; Chmielowski, B.; Jalil, J.; de la Rocha, P.; McCannel, T.A.; Ochoa, M.T.; Seja, E.; Villanueva, A.; Oseguera, D.K., et al. Dendritic cell vaccination combined with CTLA4 blockade in patients with metastatic melanoma. *Clinical cancer research : an official journal of the American Association for Cancer Research* **2009**, *15*, 6267-6276, doi:10.1158/1078-0432.CCR-09-1254.
21. Nestle, F.O.; Alijagic, S.; Gilliet, M.; Sun, Y.; Grabbe, S.; Dummer, R.; Burg, G.; Schadendorf, D. Vaccination of melanoma patients with peptide- or tumor lysate-pulsed dendritic cells. *Nature medicine* **1998**, *4*, 328-332.
22. Greene, J.M.; Schneble, E.J.; Jackson, D.O.; Hale, D.F.; Vreeland, T.J.; Flores, M.; Martin, J.; Herbert, G.S.; Hardin, M.O.; Yu, X., et al. A phase I/IIa clinical trial in stage IV melanoma of an autologous tumor-dendritic cell fusion (dendritoma) vaccine with low dose interleukin-2. *Cancer immunology, immunotherapy : CII* **2016**, *65*, 383-392, doi:10.1007/s00262-016-1809-6.
23. Salcedo, M.; Bercovici, N.; Taylor, R.; Vereecken, P.; Massicard, S.; Duriau, D.; Vernel-Pauillac, F.; Boyer, A.; Baron-Bodo, V.; Mallard, E., et al. Vaccination of melanoma patients using dendritic cells loaded with an allogeneic tumor cell lysate. *Cancer immunology, immunotherapy : CII* **2006**, *55*, 819-829, doi:10.1007/s00262-005-0078-6.
24. Rozera, C.; Cappellini, G.A.; D'Agostino, G.; Santodonato, L.; Castiello, L.; Urbani, F.; Macchia, I.; Arico, E.; Casorelli, I.; Sestili, P., et al. Intratumoral injection of IFN-alpha dendritic cells after dacarbazine activates anti-tumor immunity: results from a phase I trial in advanced melanoma. *J Transl Med* **2015**, *13*, 139, doi:10.1186/s12967-015-0473-5.
25. Thurner, B.; Haendle, I.; Roder, C.; Dieckmann, D.; Keikavoussi, P.; Jonuleit, H.; Bender, A.; Maczek, C.; Schreiner, D.; von den Driesch, P., et al. Vaccination with mage-3A1 peptide-pulsed mature, monocyte-derived dendritic cells expands specific cytotoxic T cells and induces regression of some metastases in advanced stage IV melanoma. *The Journal of experimental medicine* **1999**, *190*, 1669-1678.
26. Poschke, I.; Lovgren, T.; Adamson, L.; Nystrom, M.; Andersson, E.; Hansson, J.; Tell, R.; Masucci, G.V.; Kiessling, R. A phase I clinical trial combining dendritic cell vaccination with adoptive T cell transfer in patients with stage IV melanoma. *Cancer immunology, immunotherapy : CII* **2014**, *63*, 1061-1071, doi:10.1007/s00262-014-1575-2.
27. Schuler-Thurner, B.; Schultz, E.S.; Berger, T.G.; Weinlich, G.; Ebner, S.; Woerl, P.; Bender, A.; Feuerstein, B.; Fritsch, P.O.; Romani, N., et al. Rapid induction of tumor-specific type 1 T helper cells in metastatic melanoma patients by vaccination with mature, cryopreserved, peptide-loaded monocyte-derived dendritic cells. *The Journal of experimental medicine* **2002**, *195*, 1279-1288.

28. Fukuda, K.; Funakoshi, T.; Sakurai, T.; Nakamura, Y.; Mori, M.; Tanese, K.; Tanikawa, A.; Taguchi, J.; Fujita, T.; Okamoto, M., et al. Peptide-pulsed dendritic cell vaccine in combination with carboplatin and paclitaxel chemotherapy for stage IV melanoma. *Melanoma Res* **2017**, *27*, 326-334, doi:10.1097/CMR.0000000000000342.
29. Lesterhuis, W.J.; de Vries, I.J.; Schreibelt, G.; Lambeck, A.J.; Aarntzen, E.H.; Jacobs, J.F.; Scharenborg, N.M.; van de Rakt, M.W.; de Boer, A.J.; Croockewit, S., et al. Route of administration modulates the induction of dendritic cell vaccine-induced antigen-specific T cells in advanced melanoma patients. *Clinical cancer research : an official journal of the American Association for Cancer Research* **2011**, *17*, 5725-5735, doi:10.1158/1078-0432.CCR-11-1261.
30. Wimmers, F.; de Haas, N.; Scholzen, A.; Schreibelt, G.; Simonetti, E.; Eleveld, M.J.; Brouwers, H.M.; Beldhuis-Valkis, M.; Joosten, I.; de Jonge, M.I., et al. Monitoring of dynamic changes in Keyhole Limpet Hemocyanin (KLH)-specific B cells in KLH-vaccinated cancer patients. *Scientific reports* **2017**, *7*, 43486, doi:10.1038/srep43486.
31. Gasser, O.; Sharples, K.J.; Barrow, C.; Williams, G.M.; Bauer, E.; Wood, C.E.; Mester, B.; Dzhelali, M.; Caygill, G.; Jones, J., et al. A phase I vaccination study with dendritic cells loaded with NY-ESO-1 and alpha-galactosylceramide: induction of polyfunctional T cells in high-risk melanoma patients. *Cancer immunology, immunotherapy : CII* **2018**, *67*, 285-298, doi:10.1007/s00262-017-2085-9.
32. Bol, K.F.; Aarntzen, E.H.; Hout, F.E.; Schreibelt, G.; Creemers, J.H.; Lesterhuis, W.J.; Gerritsen, W.R.; Grunhagen, D.J.; Verhoef, C.; Punt, C.J., et al. Favorable overall survival in stage III melanoma patients after adjuvant dendritic cell vaccination. *Oncoimmunology* **2016**, *5*, e1057673, doi:10.1080/2162402X.2015.1057673.
33. Palucka, A.K.; Ueno, H.; Connolly, J.; Kerneis-Norvell, F.; Blanck, J.P.; Johnston, D.A.; Fay, J.; Banchereau, J. Dendritic cells loaded with killed allogeneic melanoma cells can induce objective clinical responses and MART-1 specific CD8+ T-cell immunity. *J Immunother* **2006**, *29*, 545-557, doi:10.1097/01.cji.0000211309.90621.8b.
34. Wilgenhof, S.; Corthals, J.; Van Nuffel, A.M.; Benteyn, D.; Heirman, C.; Bonehill, A.; Thielemans, K.; Neyns, B. Long-term clinical outcome of melanoma patients treated with messenger RNA-electroporated dendritic cell therapy following complete resection of metastases. *Cancer immunology, immunotherapy : CII* **2015**, *64*, 381-388, doi:10.1007/s00262-014-1642-8.
35. Benteyn, D.; Van Nuffel, A.M.; Wilgenhof, S.; Corthals, J.; Heirman, C.; Neyns, B.; Thielemans, K.; Bonehill, A. Characterization of CD8+ T-cell responses in the peripheral blood and skin injection sites of melanoma patients treated with mRNA electroporated autologous dendritic cells (TriMixDC-MEL). *Biomed Res Int* **2013**, *2013*, 976383, doi:10.1155/2013/976383.
36. Wilgenhof, S.; Van Nuffel, A.M.; Corthals, J.; Heirman, C.; Tuyaerts, S.; Benteyn, D.; De Coninck, A.; Van Riet, I.; Verfaillie, G.; Vandeloo, J., et al. Therapeutic vaccination with an autologous mRNA electroporated dendritic cell vaccine in patients with advanced melanoma. *J Immunother* **2011**, *34*, 448-456, doi:10.1097/CJI.0b013e31821dcb31.
37. Dannull, J.; Haley, N.R.; Archer, G.; Nair, S.; Boczkowski, D.; Harper, M.; De Rosa, N.; Pickett, N.; Mosca, P.J.; Burchette, J., et al. Melanoma immunotherapy using mature DCs expressing the constitutive proteasome. *The Journal of clinical investigation* **2013**, *123*, 3135-3145, doi:10.1172/JCI67544.
38. Mackensen, A.; Herbst, B.; Chen, J.L.; Kohler, G.; Noppen, C.; Herr, W.; Spagnoli, G.C.; Cerundolo, V.; Lindemann, A. Phase I study in melanoma patients of a vaccine with peptide-pulsed dendritic cells generated in vitro from CD34(+) hematopoietic progenitor cells. *International journal of cancer* **2000**, *86*, 385-392.
39. Banchereau, J.; Palucka, A.K.; Dhodapkar, M.; Burkeholder, S.; Taquet, N.; Rolland, A.; Taquet, S.; Coquery, S.; Wittkowski, K.M.; Bhardwaj, N., et al. Immune and clinical responses in patients with metastatic melanoma to CD34(+) progenitor-derived dendritic cell vaccine. *Cancer research* **2001**, *61*, 6451-6458.
40. Schreibelt, G.; Bol, K.F.; Westdorp, H.; Wimmers, F.; Aarntzen, E.H.; Duiveman-de Boer, T.; van de Rakt, M.W.; Scharenborg, N.M.; de Boer, A.J.; Pots, J.M., et al. Effective Clinical Responses in Metastatic Melanoma Patients after Vaccination with Primary Myeloid Dendritic Cells. *Clinical cancer research : an official journal of the American Association for Cancer Research* **2016**, *22*, 2155-2166, doi:10.1158/1078-0432.CCR-15-2205.
41. Frank, M.O.; Kaufman, J.; Parveen, S.; Blachere, N.E.; Orange, D.E.; Darnell, R.B. Dendritic cell vaccines containing lymphocytes produce improved immunogenicity in patients with cancer. *J Transl Med* **2014**, *12*, 338, doi:10.1186/s12967-014-0338-3.

42. Frank, M.O.; Kaufman, J.; Tian, S.; Suarez-Farinas, M.; Parveen, S.; Blachere, N.E.; Morris, M.J.; Slovin, S.; Scher, H.I.; Albert, M.L., et al. Harnessing naturally occurring tumor immunity: a clinical vaccine trial in prostate cancer. *PloS one* **2010**, *5*, doi:10.1371/journal.pone.0012367.
43. Podrazil, M.; Horvath, R.; Becht, E.; Rozkova, D.; Bilkova, P.; Sochorova, K.; Hromadkova, H.; Kayserova, J.; Vavrova, K.; Lastovicka, J., et al. Phase I/II clinical trial of dendritic-cell based immunotherapy (DCVAC/PCa) combined with chemotherapy in patients with metastatic, castration-resistant prostate cancer. *Oncotarget* **2015**, *6*, 18192-18205, doi:10.18632/oncotarget.4145.
44. Fucikova, J.; Podrazil, M.; Jarolim, L.; Bilkova, P.; Hensler, M.; Becht, E.; Gasova, Z.; Klouckova, J.; Kayserova, J.; Horvath, R., et al. Phase I/II trial of dendritic cell-based active cellular immunotherapy with DCVAC/PCa in patients with rising PSA after primary prostatectomy or salvage radiotherapy for the treatment of prostate cancer. *Cancer immunology, immunotherapy : CII* **2018**, *67*, 89-100, doi:10.1007/s00262-017-2068-x.
45. Kongsted, P.; Borch, T.H.; Ellebaek, E.; Iversen, T.Z.; Andersen, R.; Met, O.; Hansen, M.; Lindberg, H.; Sengelov, L.; Svane, I.M. Dendritic cell vaccination in combination with docetaxel for patients with metastatic castration-resistant prostate cancer: A randomized phase II study. *Cytotherapy* **2017**, *19*, 500-513, doi:10.1016/j.jcyt.2017.01.007.
46. Sonpavde, G.; McMannis, J.D.; Bai, Y.; Seethammagari, M.R.; Bull, J.M.C.; Hawkins, V.; Dancsak, T.K.; Lapteva, N.; Levitt, J.M.; Moseley, A., et al. Phase I trial of antigen-targeted autologous dendritic cell-based vaccine with in vivo activation of inducible CD40 for advanced prostate cancer. *Cancer immunology, immunotherapy : CII* **2017**, *66*, 1345-1357, doi:10.1007/s00262-017-2027-6.
47. Small, E.J.; Schellhammer, P.F.; Higano, C.S.; Redfern, C.H.; Nemunaitis, J.J.; Valone, F.H.; Verjee, S.S.; Jones, L.A.; Hershberg, R.M. Placebo-controlled phase III trial of immunologic therapy with sipuleucel-T (APC8015) in patients with metastatic, asymptomatic hormone refractory prostate cancer. *Journal of clinical oncology : official journal of the American Society of Clinical Oncology* **2006**, *24*, 3089-3094, doi:10.1200/JCO.2005.04.5252.
48. Kantoff, P.W.; Higano, C.S.; Shore, N.D.; Berger, E.R.; Small, E.J.; Penson, D.F.; Redfern, C.H.; Ferrari, A.C.; Dreicer, R.; Sims, R.B., et al. Sipuleucel-T immunotherapy for castration-resistant prostate cancer. *The New England journal of medicine* **2010**, *363*, 411-422, doi:10.1056/NEJMoa1001294.
49. Prue, R.L.; Vari, F.; Radford, K.J.; Tong, H.; Hardy, M.Y.; D'Rozario, R.; Waterhouse, N.J.; Rossetti, T.; Coleman, R.; Tracey, C., et al. A phase I clinical trial of CD1c (BDCA-1)+ dendritic cells pulsed with HLA-A*0201 peptides for immunotherapy of metastatic hormone refractory prostate cancer. *J Immunother* **2015**, *38*, 71-76, doi:10.1097/CJI.0000000000000063.
50. Hunn, M.K.; Bauer, E.; Wood, C.E.; Gasser, O.; Dzhelali, M.; Ancelet, L.R.; Mester, B.; Sharples, K.J.; Findlay, M.P.; Hamilton, D.A., et al. Dendritic cell vaccination combined with temozolomide retreatment: results of a phase I trial in patients with recurrent glioblastoma multiforme. *J Neurooncol* **2015**, *121*, 319-329, doi:10.1007/s11060-014-1635-7.
51. Yao, Y.; Luo, F.; Tang, C.; Chen, D.; Qin, Z.; Hua, W.; Xu, M.; Zhong, P.; Yu, S.; Chen, D., et al. Molecular subgroups and B7-H4 expression levels predict responses to dendritic cell vaccines in glioblastoma: an exploratory randomized phase II clinical trial. *Cancer immunology, immunotherapy : CII* **2018**, *67*, 1777-1788, doi:10.1007/s00262-018-2232-y.
52. Phuphanich, S.; Wheeler, C.J.; Rudnick, J.D.; Mazer, M.; Wang, H.; Nuno, M.A.; Richardson, J.E.; Fan, X.; Ji, J.; Chu, R.M., et al. Phase I trial of a multi-epitope-pulsed dendritic cell vaccine for patients with newly diagnosed glioblastoma. *Cancer immunology, immunotherapy : CII* **2013**, *62*, 125-135, doi:10.1007/s00262-012-1319-0.
53. Reap, E.A.; Suryadevara, C.M.; Batich, K.A.; Sanchez-Perez, L.; Archer, G.E.; Schmittling, R.J.; Norberg, P.K.; Herndon, J.E., 2nd; Healy, P.; Congdon, K.L., et al. Dendritic Cells Enhance Polyfunctionality of Adoptively Transferred T Cells That Target Cytomegalovirus in Glioblastoma. *Cancer research* **2018**, *78*, 256-264, doi:10.1158/0008-5472.CAN-17-0469.
54. Antonopoulos, M.; SW, V.A.N.G.; Dionysiou, D.; Graf, N.; Stamatakos, G. Immune Phenotype Correlates With Survival in Patients With GBM Treated With Standard Temozolomide-based Therapy and Immunotherapy. *Anticancer research* **2019**, *39*, 2043-2051, doi:10.21873/anticanres.13315.
55. Ardon, H.; Van Gool, S.W.; Verschuere, T.; Maes, W.; Fieuws, S.; Sciot, R.; Wilms, G.; Demaerel, P.; Goffin, J.; Van Calenbergh, F., et al. Integration of autologous dendritic cell-based immunotherapy in the standard of care treatment for patients with newly diagnosed

glioblastoma: results of the HGG-2006 phase I/II trial. *Cancer immunology, immunotherapy : CII* **2012**, *61*, 2033-2044, doi:10.1007/s00262-012-1261-1.
56. Fadul, C.E.; Fisher, J.L.; Hampton, T.H.; Lallana, E.C.; Li, Z.; Gui, J.; Szczepiorkowski, Z.M.; Tosteson, T.D.; Rhodes, C.H.; Wishart, H.A., et al. Immune response in patients with newly diagnosed glioblastoma multiforme treated with intranodal autologous tumor lysate-dendritic cell vaccination after radiation chemotherapy. *J Immunother* **2011**, *34*, 382-389, doi:10.1097/CJI.0b013e318215e300.
57. Inoges, S.; Tejada, S.; de Cerio, A.L.; Gallego Perez-Larraya, J.; Espinos, J.; Idoate, M.A.; Dominguez, P.D.; de Eulate, R.G.; Aristu, J.; Bendandi, M., et al. A phase II trial of autologous dendritic cell vaccination and radiochemotherapy following fluorescence-guided surgery in newly diagnosed glioblastoma patients. *J Transl Med* **2017**, *15*, 104, doi:10.1186/s12967-017-1202-z.
58. Mitchell, D.A.; Batich, K.A.; Gunn, M.D.; Huang, M.N.; Sanchez-Perez, L.; Nair, S.K.; Congdon, K.L.; Reap, E.A.; Archer, G.E.; Desjardins, A., et al. Tetanus toxoid and CCL3 improve dendritic cell vaccines in mice and glioblastoma patients. *Nature* **2015**, *519*, 366-369, doi:10.1038/nature14320.
59. Batich, K.A.; Reap, E.A.; Archer, G.E.; Sanchez-Perez, L.; Nair, S.K.; Schmittling, R.J.; Norberg, P.; Xie, W.; Herndon, J.E., 2nd; Healy, P., et al. Long-term Survival in Glioblastoma with Cytomegalovirus pp65-Targeted Vaccination. *Clinical cancer research : an official journal of the American Association for Cancer Research* **2017**, *23*, 1898-1909, doi:10.1158/1078-0432.CCR-16-2057.
60. Hirschowitz, E.A.; Foody, T.; Hidalgo, G.E.; Yannelli, J.R. Immunization of NSCLC patients with antigen-pulsed immature autologous dendritic cells. *Lung cancer* **2007**, *57*, 365-372, doi:10.1016/j.lungcan.2007.04.002.
61. Hirschowitz, E.A.; Foody, T.; Kryscio, R.; Dickson, L.; Sturgill, J.; Yannelli, J. Autologous dendritic cell vaccines for non-small-cell lung cancer. *Journal of clinical oncology : official journal of the American Society of Clinical Oncology* **2004**, *22*, 2808-2815, doi:10.1200/JCO.2004.01.074.
62. Lee, J.M.; Lee, M.H.; Garon, E.; Goldman, J.W.; Salehi-Rad, R.; Baratelli, F.E.; Schaue, D.; Wang, G.; Rosen, F.; Yanagawa, J., et al. Phase I Trial of Intratumoral Injection of CCL21 Gene-Modified Dendritic Cells in Lung Cancer Elicits Tumor-Specific Immune Responses and CD8(+) T-cell Infiltration. *Clinical cancer research : an official journal of the American Association for Cancer Research* **2017**, *23*, 4556-4568, doi:10.1158/1078-0432.CCR-16-2821.
63. Zhang, L.; Xu, Y.; Shen, J.; He, F.; Zhang, D.; Chen, Z.; Duan, Y.; Sun, J. Feasibility study of DCs/CIKs combined with thoracic radiotherapy for patients with locally advanced or metastatic non-small-cell lung cancer. *Radiat Oncol* **2016**, *11*, 60, doi:10.1186/s13014-016-0635-5.
64. Zhao, P.; Bu, X.; Wei, X.; Sun, W.; Xie, X.; Li, C.; Guo, Q.; Zhu, D.; Wei, X.; Gao, D. Dendritic cell immunotherapy combined with cytokine-induced killer cells promotes skewing toward Th2 cytokine profile in patients with metastatic non-small cell lung cancer. *Int Immunopharmacol* **2015**, *25*, 450-456, doi:10.1016/j.intimp.2015.02.010.
65. Ge, C.; Li, R.; Song, H.; Geng, T.; Yang, J.; Tan, Q.; Song, L.; Wang, Y.; Xue, Y.; Li, Z., et al. Phase I clinical trial of a novel autologous modified-DC vaccine in patients with resected NSCLC. *BMC Cancer* **2017**, *17*, 884, doi:10.1186/s12885-017-3859-3.
66. Kimura, H.; Matsui, Y.; Ishikawa, A.; Nakajima, T.; Yoshino, M.; Sakairi, Y. Randomized controlled phase III trial of adjuvant chemo-immunotherapy with activated killer T cells and dendritic cells in patients with resected primary lung cancer. *Cancer immunology, immunotherapy : CII* **2015**, *64*, 51-59, doi:10.1007/s00262-014-1613-0.
67. Chiappori, A.A.; Williams, C.C.; Gray, J.E.; Tanvetyanon, T.; Haura, E.B.; Creelan, B.C.; Thapa, R.; Chen, D.T.; Simon, G.R.; Bepler, G., et al. Randomized-controlled phase II trial of salvage chemotherapy after immunization with a TP53-transfected dendritic cell-based vaccine (Ad.p53-DC) in patients with recurrent small cell lung cancer. *Cancer immunology, immunotherapy : CII* **2019**, *68*, 517-527, doi:10.1007/s00262-018-2287-9.
68. Wang, X.; Bayer, M.E.; Chen, X.; Fredrickson, C.; Cornforth, A.N.; Liang, G.; Cannon, J.; He, J.; Fu, Q.; Liu, J., et al. Phase I trial of active specific immunotherapy with autologous dendritic cells pulsed with autologous irradiated tumor stem cells in hepatitis B-positive patients with hepatocellular carcinoma. *J Surg Oncol* **2015**, *111*, 862-867, doi:10.1002/jso.23897.
69. Maeda, Y.; Yoshimura, K.; Matsui, H.; Shindo, Y.; Tamesa, T.; Tokumitsu, Y.; Hashimoto, N.; Tokuhisa, Y.; Sakamoto, K.; Sakai, K., et al. Dendritic cells transfected with heat-shock protein 70 messenger RNA for patients with hepatitis C virus-related hepatocellular carcinoma: a phase 1 dose escalation clinical trial. *Cancer immunology, immunotherapy : CII* **2015**, *64*, 1047-1056, doi:10.1007/s00262-015-1709-1.

70. Lee, J.H.; Lee, Y.; Lee, M.; Heo, M.K.; Song, J.S.; Kim, K.H.; Lee, H.; Yi, N.J.; Lee, K.W.; Suh, K.S., et al. A phase I/IIa study of adjuvant immunotherapy with tumour antigen-pulsed dendritic cells in patients with hepatocellular carcinoma. *British journal of cancer* **2015**, *113*, 1666-1676, doi:10.1038/bjc.2015.430.
71. Mehrotra, S.; Britten, C.D.; Chin, S.; Garrett-Mayer, E.; Cloud, C.A.; Li, M.; Scurti, G.; Salem, M.L.; Nelson, M.H.; Thomas, M.B., et al. Vaccination with poly(IC:LC) and peptide-pulsed autologous dendritic cells in patients with pancreatic cancer. *J Hematol Oncol* **2017**, *10*, 82, doi:10.1186/s13045-017-0459-2.
72. Tsukinaga, S.; Kajihara, M.; Takakura, K.; Ito, Z.; Kanai, T.; Saito, K.; Takami, S.; Kobayashi, H.; Matsumoto, Y.; Odahara, S., et al. Prognostic significance of plasma interleukin-6/-8 in pancreatic cancer patients receiving chemoimmunotherapy. *World J Gastroenterol* **2015**, *21*, 11168-11178, doi:10.3748/wjg.v21.i39.11168.
73. Mayanagi, S.; Kitago, M.; Sakurai, T.; Matsuda, T.; Fujita, T.; Higuchi, H.; Taguchi, J.; Takeuchi, H.; Itano, O.; Aiura, K., et al. Phase I pilot study of Wilms tumor gene 1 peptide-pulsed dendritic cell vaccination combined with gemcitabine in pancreatic cancer. *Cancer science* **2015**, *106*, 397-406, doi:10.1111/cas.12621.
74. Jiang, N.; Qiao, G.; Wang, X.; Morse, M.A.; Gwin, W.R.; Zhou, L.; Song, Y.; Zhao, Y.; Chen, F.; Zhou, X., et al. Dendritic Cell/Cytokine-Induced Killer Cell Immunotherapy Combined with S-1 in Patients with Advanced Pancreatic Cancer: A Prospective Study. *Clinical cancer research : an official journal of the American Association for Cancer Research* **2017**, *23*, 5066-5073, doi:10.1158/1078-0432.CCR-17-0492.
75. Ho, V.T.; Kim, H.T.; Kao, G.; Cutler, C.; Levine, J.; Rosenblatt, J.; Joyce, R.; Antin, J.H.; Soiffer, R.J.; Ritz, J., et al. Sequential infusion of donor-derived dendritic cells with donor lymphocyte infusion for relapsed hematologic cancers after allogeneic hematopoietic stem cell transplantation. *Am J Hematol* **2014**, *89*, 1092-1096, doi:10.1002/ajh.23825.
76. Kolstad, A.; Kumari, S.; Walczak, M.; Madsbu, U.; Hagtvedt, T.; Bogsrud, T.V.; Kvalheim, G.; Holte, H.; Aurlien, E.; Delabie, J., et al. Sequential intranodal immunotherapy induces antitumor immunity and correlated regression of disseminated follicular lymphoma. *Blood* **2015**, *125*, 82-89, doi:10.1182/blood-2014-07-592162.
77. Qiu, Y.; Yun, M.M.; Dong, X.; Xu, M.; Zhao, R.; Han, X.; Zhou, E.; Yun, F.; Su, W.; Liu, C., et al. Combination of cytokine-induced killer and dendritic cells pulsed with antigenic alpha-1,3-galactosyl epitope-enhanced lymphoma cell membrane for effective B-cell lymphoma immunotherapy. *Cytotherapy* **2016**, *18*, 91-98, doi:10.1016/j.jcyt.2015.09.012.
78. Anguille, S.; Van de Velde, A.L.; Smits, E.L.; Van Tendeloo, V.F.; Juliusson, G.; Cools, N.; Nijs, G.; Stein, B.; Lion, E.; Van Driessche, A., et al. Dendritic cell vaccination as postremission treatment to prevent or delay relapse in acute myeloid leukemia. *Blood* **2017**, *130*, 1713-1721, doi:10.1182/blood-2017-04-780155.
79. Khoury, H.J.; Collins, R.H., Jr.; Blum, W.; Stiff, P.S.; Elias, L.; Lebkowski, J.S.; Reddy, A.; Nishimoto, K.P.; Sen, D.; Wirth, E.D., 3rd, et al. Immune responses and long-term disease recurrence status after telomerase-based dendritic cell immunotherapy in patients with acute myeloid leukemia. *Cancer* **2017**, *123*, 3061-3072, doi:10.1002/cncr.30696.
80. van de Loosdrecht, A.A.; van Wetering, S.; Santegoets, S.; Singh, S.K.; Eeltink, C.M.; den Hartog, Y.; Koppes, M.; Kaspers, J.; Ossenkoppele, G.J.; Kruisbeek, A.M., et al. A novel allogeneic off-the-shelf dendritic cell vaccine for post-remission treatment of elderly patients with acute myeloid leukemia. *Cancer immunology, immunotherapy : CII* **2018**, *67*, 1505-1518, doi:10.1007/s00262-018-2198-9.
81. Oostvogels, R.; Kneppers, E.; Minnema, M.C.; Doorn, R.C.; Franssen, L.E.; Aarts, T.; Emmelot, M.E.; Spierings, E.; Slaper-Cortenbach, I.; Westinga, K., et al. Efficacy of host-dendritic cell vaccinations with or without minor histocompatibility antigen loading, combined with donor lymphocyte infusion in multiple myeloma patients. *Bone marrow transplantation* **2017**, *52*, 228-237, doi:10.1038/bmt.2016.250.
82. Franssen, L.E.; Roeven, M.W.H.; Hobo, W.; Doorn, R.; Oostvogels, R.; Falkenburg, J.H.F.; van de Donk, N.W.; Kester, M.G.D.; Fredrix, H.; Westinga, K., et al. A phase I/II minor histocompatibility antigen-loaded dendritic cell vaccination trial to safely improve the efficacy of donor lymphocyte infusions in myeloma. *Bone Marrow Transplant* **2017**, *52*, 1378-1383, doi:10.1038/bmt.2017.118.
83. Gray, H.J.; Benigno, B.; Berek, J.; Chang, J.; Mason, J.; Mileshkin, L.; Mitchell, P.; Moradi, M.; Recio, F.O.; Michener, C.M., et al. Progression-free and overall survival in ovarian cancer patients treated with CVac, a mucin 1 dendritic cell therapy in a randomized phase 2 trial. *J Immunother Cancer* **2016**, *4*, 34, doi:10.1186/s40425-016-0137-x.

84. Baek, S.; Kim, Y.M.; Kim, S.B.; Kim, C.S.; Kwon, S.W.; Kim, Y.; Kim, H.; Lee, H. Therapeutic DC vaccination with IL-2 as a consolidation therapy for ovarian cancer patients: a phase I/II trial. *Cell Mol Immunol* **2015**, *12*, 87-95, doi:10.1038/cmi.2014.40.
85. Ferrara, A.; Nonn, M.; Sehr, P.; Schreckenberger, C.; Pawlita, M.; Durst, M.; Schneider, A.; Kaufmann, A.M. Dendritic cell-based tumor vaccine for cervical cancer II: results of a clinical pilot study in 15 individual patients. *J Cancer Res Clin Oncol* **2003**, *129*, 521-530, doi:10.1007/s00432-003-0463-5.
86. Koski, G.K.; Koldovsky, U.; Xu, S.; Mick, R.; Sharma, A.; Fitzpatrick, E.; Weinstein, S.; Nisenbaum, H.; Levine, B.L.; Fox, K., et al. A novel dendritic cell-based immunization approach for the induction of durable Th1-polarized anti-HER-2/neu responses in women with early breast cancer. *J Immunother* **2012**, *35*, 54-65, doi:10.1097/CJI.0b013e318235f512.
87. Lowenfeld, L.; Mick, R.; Datta, J.; Xu, S.; Fitzpatrick, E.; Fisher, C.S.; Fox, K.R.; DeMichele, A.; Zhang, P.J.; Weinstein, S.P., et al. Dendritic Cell Vaccination Enhances Immune Responses and Induces Regression of HER2(pos) DCIS Independent of Route: Results of Randomized Selection Design Trial. *Clinical cancer research : an official journal of the American Association for Cancer Research* **2017**, *23*, 2961-2971, doi:10.1158/1078-0432.CCR-16-1924.
88. Zhao, X.; Zhang, Z.; Li, H.; Huang, J.; Yang, S.; Xie, T.; Huang, L.; Yue, D.; Xu, L.; Wang, L., et al. Cytokine induced killer cell-based immunotherapies in patients with different stages of renal cell carcinoma. *Cancer Lett* **2015**, *362*, 192-198, doi:10.1016/j.canlet.2015.03.043.
89. Laurell, A.; Lonnemark, M.; Brekkan, E.; Magnusson, A.; Tolf, A.; Wallgren, A.C.; Andersson, B.; Adamson, L.; Kiessling, R.; Karlsson-Parra, A. Intratumorally injected pro-inflammatory allogeneic dendritic cells as immune enhancers: a first-in-human study in unfavourable risk patients with metastatic renal cell carcinoma. *Journal for immunotherapy of cancer* **2017**, *5*, 52, doi:10.1186/s40425-017-0255-0.
90. Krishnadas, D.K.; Shusterman, S.; Bai, F.; Diller, L.; Sullivan, J.E.; Cheerva, A.C.; George, R.E.; Lucas, K.G. A phase I trial combining decitabine/dendritic cell vaccine targeting MAGE-A1, MAGE-A3 and NY-ESO-1 for children with relapsed or therapy-refractory neuroblastoma and sarcoma. *Cancer immunology, immunotherapy : CII* **2015**, *64*, 1251-1260, doi:10.1007/s00262-015-1731-3.
91. Miwa, S.; Nishida, H.; Tanzawa, Y.; Takeuchi, A.; Hayashi, K.; Yamamoto, N.; Mizukoshi, E.; Nakamoto, Y.; Kaneko, S.; Tsuchiya, H. Phase 1/2 study of immunotherapy with dendritic cells pulsed with autologous tumor lysate in patients with refractory bone and soft tissue sarcoma. *Cancer* **2017**, *123*, 1576-1584, doi:10.1002/cncr.30606.
92. Hijikata, Y.; Okazaki, T.; Tanaka, Y.; Murahashi, M.; Yamada, Y.; Yamada, K.; Takahashi, A.; Inoue, H.; Kishimoto, J.; Nakanishi, Y., et al. A phase I clinical trial of RNF43 peptide-related immune cell therapy combined with low-dose cyclophosphamide in patients with advanced solid tumors. *PloS one* **2018**, *13*, e0187878, doi:10.1371/journal.pone.0187878.
